# Metabolic profiling and antioxidant activity of fenugreek seeds cultivars ‘Giza 2’ and ‘Giza 30’ compared to other geographically-related seeds

**DOI:** 10.1016/j.fochx.2024.101819

**Published:** 2024-09-07

**Authors:** Reham Hassan Mekky, Essam Abdel-Sattar, Maha-Hamadien Abdulla, Antonio Segura-Carretero, Khayal Al-Khayal, Wagdy M. Eldehna, María del Mar Contreras

**Affiliations:** a Department of Pharmacognosy, Faculty of Pharmacy, Egyptian Russian University, Badr City, Cairo-Suez Road, 11829 Cairo, Egypt; b Research and Development Functional Food Centre (CIDAF), Bioregiόn Building, Health Science Technological Park, Avenida del Conocimiento s/n, 18016 Granada, Spain; c Department of Pharmacognosy, Faculty of Pharmacy, Cairo University, El Kasr El-Aini Street, 11562 Cairo, Egypt; d Department of Surgery, College of Medicine, King Saud University, Riyadh, Saudi Arabia; e Department of Analytical Chemistry, Faculty of Sciences, University of Granada, Avenida Fuentenueva s/n, 18071 Granada, Spain; f Department of Pharmaceutical Chemistry, Faculty of Pharmacy, Kafrelsheikh University, Kafrelsheikh, P.O. Box 33516, Egypt; g Department of Pharmaceutical Chemistry, Faculty of Pharmacy, Pharos University in Alexandria; Canal El Mahmoudia St., Alexandria 21648, Egypt; h Department of Chemical, Environmental and Materials Engineering, Universidad de Jaén, Campus Las Lagunillas, 23071 Jaén, Spain

**Keywords:** Fenugreek, Flavonoids, Furostanol saponins, Metabolomics, Multivariate data analysis

## Abstract

This study addresses a comparative comprehensive metabolic profiling of two Egyptian cultivars of fenugreek (*Trigonella foenum-graecum* L.) seeds ‘Giza 2’ and ‘Giza 30’ via RP-HPLC-DAD-QTOF-MS and MS/MS. Briefly, 126 metabolites were detected in the samples under investigation, being classified into hydroxybenzoic acids (8), hydroxycinnamic acids (7), flavonoids (49 with a predominancy of flavones in particular apigenin derivatives), coumarins (1), furostanol saponins (17), alkaloids (2), amino acids (11), peptides (2), jasmonates (6), nucleosides (30), organic acids (16), terpenoids (1), and sugars (3). In addition, the total phenolic content and antioxidant activity were determined and compared with other geographically related seeds (chickpea Giza-1, sesame Giza-32, and linseed, Giza-10), showing slight differences among them but higher values than the other geographically related seeds that were segregated from them upon chemometric analysis. This is the first comprehensive metabolic profiling of these cultivars, presenting an initial account of some metabolites found in Fabaceae, such as apigenin di *C* pentoside, with a significant occurrence of biologically active furostanol saponins. It gives a prospect of fenugreeks richness of bioactive metabolites as an essential functional food that could add value to the food and nutraceutical industries' sustainability.

## Highlights

• A comprehensive metabolic profiling of fenugreek seed cultivars ‘Giza 2’ and ‘Giza 30’ was conducted.
• In total, 126 metabolites were characterized by analyzing two cultivars.
• Flavonoids and furostanol saponins were the main phytochemical classes.
• ‘Giza 2’ and ‘Giza 30’ fenugreek seeds exhibit higher antioxidant activity than other regional seeds.
• A prospect for the importance of fenugreeks in the food and nutraceuticals industry.

## Graphical abstract

**Figure:**
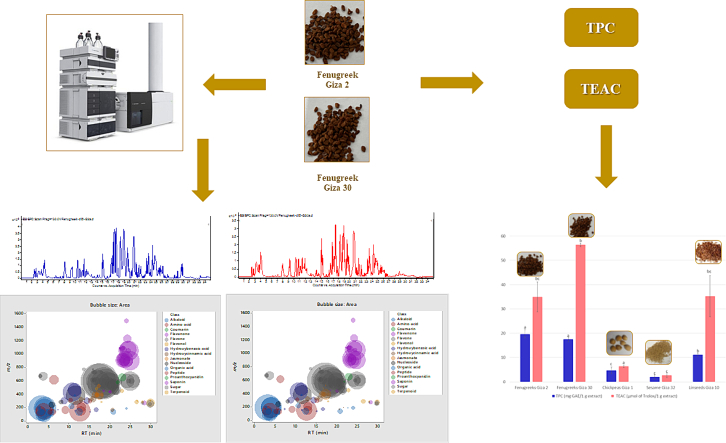

## 1 Introduction

Fenugreek seeds (*Trigonella foenum-graecum* L., family Fabaceae) are one of the ancient spices whose cultivation dates to 4000 BCE It is native to the Mediterranean region, but India is the main producer today. This plant was used by the Ancient Egyptians where the first recorded use of fenugreeks was around 1500 BCE in Egyptian papyrus (Snehlata & Payal, 2012). Traditionally, fenugreeks were employed in Ancient Egypt, China, Europe, and Ayurvedic medicine in inflammatory, respiratory, and gastrointestinal disorders, among others (Aboelsoud, 2010; Singh, Yadav, Kumar, & Narashiman, 2022; Yao et al., 2020). Fenugreek seeds are a rich source of dietary proteins, carbohydrates, dietary fibers, lipids, minerals, and trace elements (Singh et al., 2022). Furthermore, fenugreeks contain several phytochemical classes, such as phenolic compounds (Farag, Rasheed, Kropf, & Heiss, 2016; Singh et al., 2022) steroidal saponins (Murakami, Kishi, Matsuda, & YOSHIKAWA, 2000; Pang et al., 2012) fatty acids and phosphatidylethanolamines (Chatterjee, Variyar, & Sharma, 2010; Farag et al., 2016) polysaccharides, and alkaloids (Singh et al., 2022). Fenugreek seeds and their extracts possess a wide range of biological activities, which can be credited to their bioactive phytochemicals (Singh et al., 2022; Yao et al., 2020). For example, the antidiabetic activity of fenugreek seeds' aqueous extract and the galactomannan-rich fraction in rats-diabetic models has been proven to attenuate pancreatic and liver damage as well as diabetic nephropathy (Alsuliam et al., 2022). Moreover, another study figured out that swimming training accompanied by the administration of hydroalcoholic extract of fenugreek seed to diabetic rats enhanced synergistically glycemic indices and the lipid profile (Hosseini, Hamzavi, Safarzadeh, & Salehi, 2023). Besides, many clinical trials investigated their effect on diabetes, hyperlipidemia, neurological disorders, and reproductive health (Singh et al., 2022). (Yao et al., 2020). Overall, the health benefits properties of fenugreek seeds are highly recognized, resulting in the world trading of fenugreek seeds as raw powder, extracts, and bioactive components as dietary supplements (Yao et al., 2020). Moreover, recent works support the use of fenugreek seeds as a feed additive to improve the growth performance of animals (Abdel-Wareth, Elkhateeb, Ismail, Ghazalah, & Lohakare, 2021) and as an immune stimulant for fish (Moustafa et al., 2020).

Among the analytical techniques to analyze fenugreeks, high-performance-liquid chromatography (HPLC) coupled to ultraviolet/visible or diode array (DAD) detectors and/or mass spectrometry (MS) and tandem (MS/MS) (Farag et al., 2016; Gonda et al., 2023) and gas chromatography (Farag et al., 2016). The phytochemical content of fenugreek seeds is complex and affected by the cultivar, germination, and cultivation type, among other factors (Singh et al., 2022). Therefore, it is crucial to provide approaches that enable multi-compound analysis, either alone or through various complementary technologies.

This work aims to conduct a comparative untargeted metabolic profiling on fenugreek seeds that enables characterizing compounds from different classes that are relevant to explain the bioactive potential employing reversed-phase (RP)-HPLC coupled to diode array detection (DAD) and quadrupole-time-of-flight (QTOF)-mass spectrometry on two Egyptian cultivars (‘Giza 2’, ‘G2’ and ‘Giza 30’, ‘G30’). Besides, the total phenolic content and antioxidant activity were determined and compared to other seed types. This article provides a comprehensive multi-compound analysis and characterization of fenugreek seeds, which is helpful in fenugreek metabolomics for characterization, quality control, pharmacology, authentication, etc.

## 2 Materials and methods

### 2.1 Chemicals and samples

The solvents used were analytical and HPLC-MS grade from Fisher Chemicals (ThermoFisher, Waltham, MA, USA). All standards were from Sigma-Aldrich (St. Louis, MO, USA) except for Kaempferide (Extrasynthese, Genay, France).

The fenugreek seeds (cultivars ‘Giza 2’ and ‘Giza 30’) were provided and authenticated by Agriculture Engineer Nadia Abdel-Azim, Egyptian Ministry of Agriculture and Land Reclamation, Giza, Egypt. Prior to extraction, the seeds were ground with an Ultra Centrifugal Mill ZM 200 (sieve around 1 mm), Retsch (Haan, Germany).

### 2.2 Extraction

Fenugreek seeds powder was extracted at room temperature in triplicate according to (Mekky et al., 2015), where it was homogenized with 50 % (v/v) methanol in water at 0.5 g/25 mL (Ultra-Turrax Ika T18 basic, Ika-Werke GmbH & Co. KG; Staufen, Germany), 10 min sonicated (B3510 sonicator; Branson, Danbury, CT, USA), agitated with magnetic stirrer (Agimatic-N Jp Selecta, Barcelona, Spain) for an hour, and centrifuged at 7155*g* for 15 min. The sediments were re-extracted with 25 mL 70 % (v/v) acetone in water and centrifuged. The supernatants were combined, and the solvent mixture evaporated with a Rotavapor R-200 and a water bath at 38 °C (Büchi Labortechnik, AG, Switzerland). Evaporated extracts were reconstituted in 2 mL 80 % (*v*/v) methanol and subjected to (0.45 μm regenerated cellulose syringe filters) for analysis. Other seeds (chickpeas, sesame, and linseeds) were extracted using similar conditions as in this work, but sesame and linseeds were first defatted (Mekky, Abdel-Sattar, Segura-Carretero, & Contreras, 2019; Mekky, Abdel-Sattar, Segura-Carretero, & Contreras, 2022).

### 2.3 Analysis by RP-HPLC-DAD-ESI-QTOF-MS and -MS/MS

An Agilent 1200 series rapid resolution (Santa Clara, CA, USA) equipped with a binary pump, an autosampler, and ultraviolet detector was employed for analysis using a core-shell Halo C18 analytical column (150 mm × 4.6 mm, 2.7 μm particle size) for separation. A 6540 Agilent Ultra-High-Definition (UHD) Accurate-Mass Q-TOF LC/MS (Palo Alto, CA, USA) equipped with an electrospray (ESI) interface and set in the negative ionization mode was coupled to the separation system (Mekky et al., 2022; Saeed et al., 2024; Tej et al., 2024). Two mobile phases consisting of acidified water (0.5 % acetic acid, v/v) (phase A) and acetonitrile (phase B) were utilized for gradient elution with a constant flow rate of 0.5 mL/min according to (Mekky et al., 2022). The injection volume was 8 μL, and the replicates of extracts were analyzed.

The operating conditions briefly were: drying nitrogen gas temperature 325 °C with flow of 10 L/min; nebulizer pressure 20 psig; sheath gas temperature 400 °C with flow 12 L/min; capillary voltage 4000 V, nozzle voltage 500 V, fragmentor voltage 130 V, skimmer voltage 45 V, octapole radiofrequency voltage 750 V. Data acquisition (2.5 Hz) in profile mode was governed via MassHunter Workstation software (Agilent technologies). The spectra were acquired in negative-ion mode over a mass-to-charge (*m/z*) range from 70 to 1500. The detection window was set to 100 ppm. Reference mass correction on each sample was performed with a continuous infusion of Agilent TOF biopolymer analysis mixture containing trifluoroacetic acid ammonium salt (*m/z* 112.9856) and hexakis (1H, 1H, 3H-tetrafluoropropoxy) phosphazine (*m/z* 1033.9881 corresponding to the trifluoroacetic acid ammonium salt adduct).

MassHunter Qualitative Analysis B.06.00 (Agilent Technologies) was used for Data analysis. For metabolites profiling, each candidate formula was generated (± 5 ppm accuracy) taking into account RT, UV, MS/MS spectra, isotopic pattern, and MS score. The characterized metabolites were compared with available standards whenever possible (Mekky, Abdel-Sattar, Segura-Carretero, & Contreras, 2021; Mekky et al., 2015).

### 2.4 Total phenol content (TPC) and Trolox equivalent antioxidant activity (TEAC)

TPC was determined in triplicate by colorimetrically by Folin–Ciocalteu reagent by (Singleton & Rossi, 1965) with modification (Hassanin et al., 2024; Mekky et al., 2022) with a microplate reader (Synergy Mx Monochromator-Based Multi-Mode, Bio-Tek Instruments Inc., Winooski, VT, USA) at a λ_max_ of 760 nm after incubation for 2 h in the dark. Serially diluted gallic acid was employed for a calibration curve, and the results were as gallic acid equivalent.

TEAC was conducted using the same microplate reader (Mekky et al., 2015; Mekky et al., 2022). This assay is based on the reduction of the cation of the radical of 2,2′-azinobis-(3-ethylbenzothiazoline-6-sulfonate) (ABTS) upon reacting ABTS stock solution (2.45 mM potassium persulfate). The mixture was incubated in the dark at room temperature for 24 h. The stock solution was diluted with water till reaching an absorbance value of 0.70 (±0.03) at 734 nm. Then, 300 μL of diluted solution and 30 μL of the extract were mixed and measured at 734 nm (25 °C). The absorbance reading was compared to a standard calibration curve of Trolox.

### 2.5 Statistical analysis

Data were expressed as mean ± standard deviation (*n* = 3). Microsoft Excel 365 (Redmond, WA, USA) and Minitab 17 (Minitab, Inc., USA) were employed for statistical analysis. One-way analysis of variance (ANOVA) was performed to assess statistical differences at 95 % using the LSD posthoc test and the software IBM SPSS Statistics 22 (Armonk, NY, USA). XLSTAT PREMIUM (Lumivero, Denver, CO, USA) was employed for multivariate data analysis.

## 3 Results and discussion

### 3.1 Metabolic profiling of the Egyptian cultivars of fenugreek

**Fig. 1:**
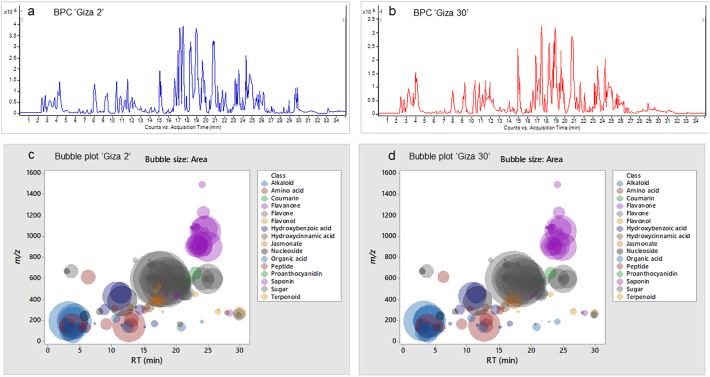
(BPCs) of (a) ‘G2’ and (b) ‘G30’, and bubble plots of *m/z* vs the RT in relation to metabolites classes (c) ‘G2’ and (d) ‘G30’.

The metabolic profiling of (‘G2’) and (‘G30’) fenugreek cultivars via RP-HPLC-DAD-ESI-QTOF-MS and MS/MS in negative mode revealed 124 and 119 compounds, respectively. Figs. 1a and 1b exhibit the base peak chromatograms (BPC) of ‘G2’ and ‘G30’, respectively. The characterized compounds included phenolic derivatives (65, Tables 1, S1) *viz*., 8 hydroxybenzoic acids, 7 hydroxycinnamic acids, 36 flavones, 7 flavonols, 5 flavanones, a coumarin and a proanthocyanidin; and non-phenolic compounds (61, Tables 2, S2) namely 17 saponins, 2 alkaloids, 10 amino acids, 3 peptides, 3 nucleosides, 6 jasmonates, 16 organic acids, 3 sugars and a terpenoid. The metabolites were identified based on retention time, observed masses, molecular formulas, fragments, UV absorption maxima, and comparison with standards and literature data (Farag et al., 2016; Hassanin et al., 2024; KNApSAcK-Core-System, 2024; Mekky et al., 2020; Mekky et al., 2021; Reaxys, 2024; Singh et al., 2022). Figs. 1c and 1d describe the different classes of the characterized metabolites in ‘G2’ and ‘G30’ using bubble plots, where the compounds are characterized by their classes, peak areas, and retention times. In this context, Table 1, Table 2 highlight the phenolic and non-phenolic compounds in ‘G2’ and ‘G30’.

**Table 1:**
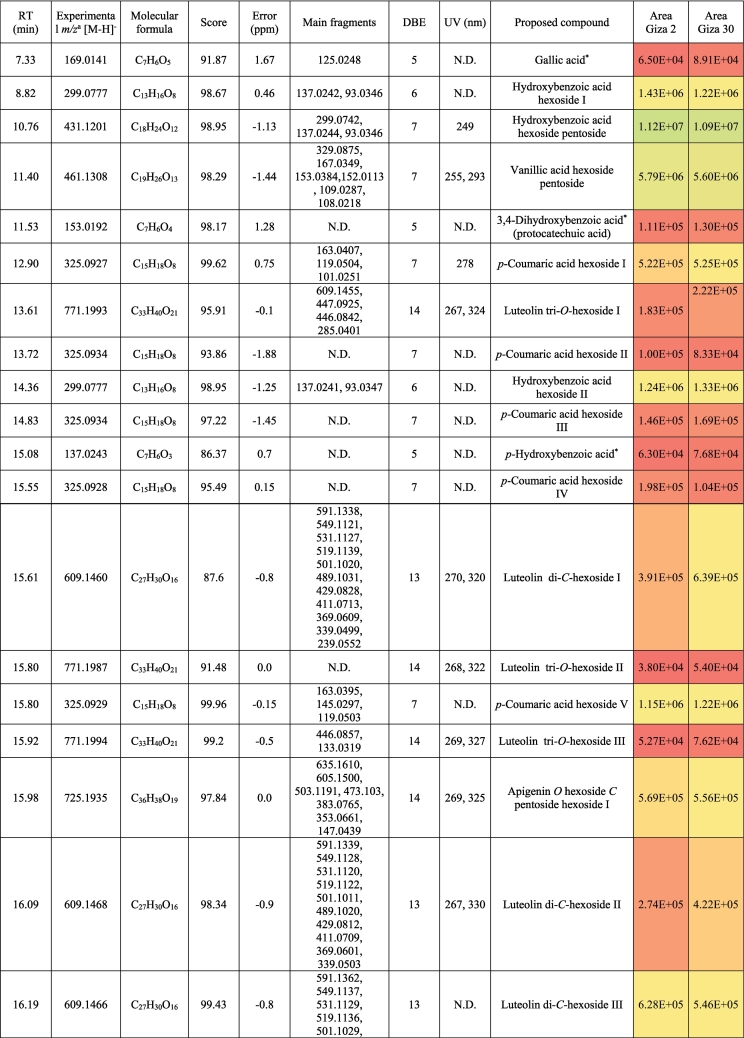

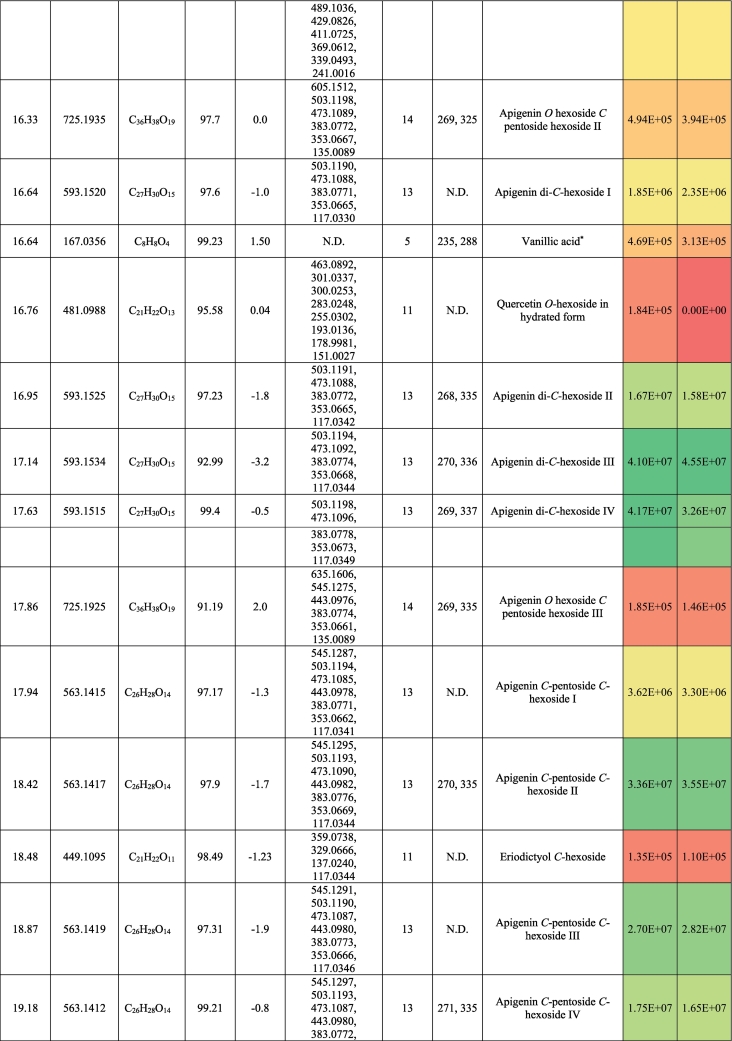

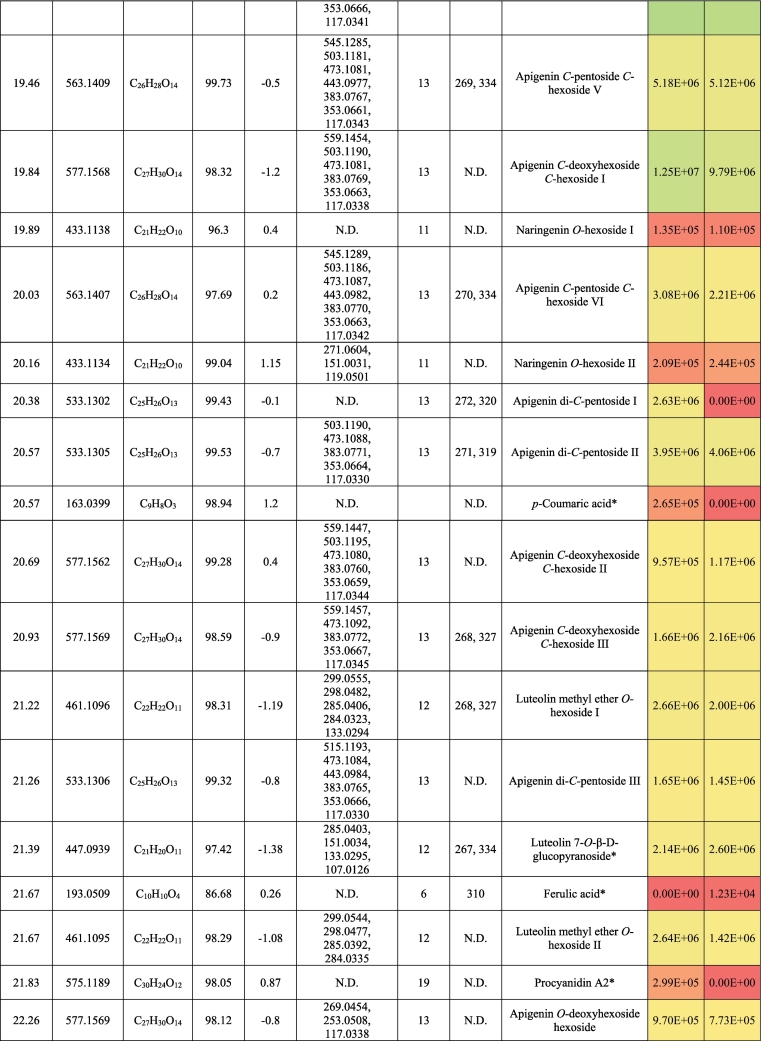

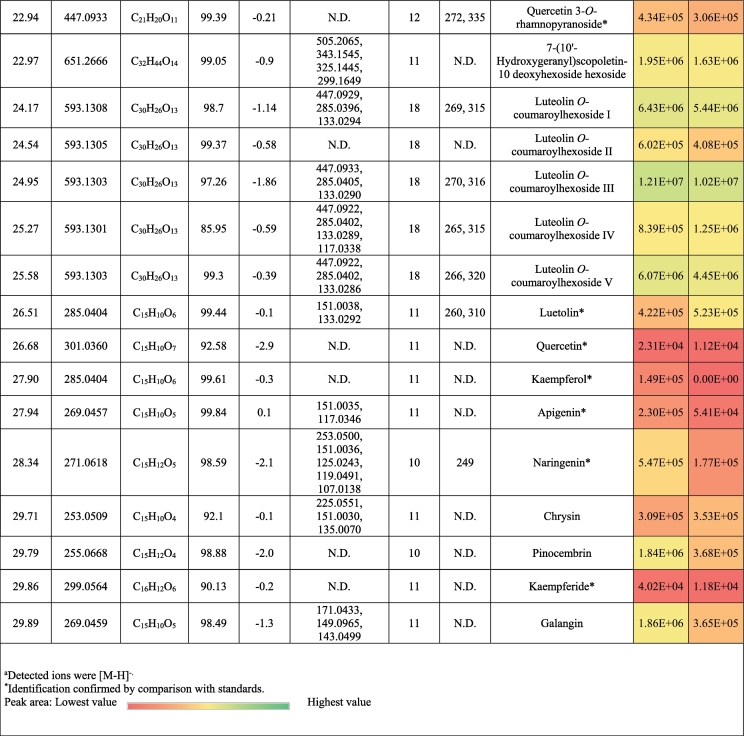
Phenolic compounds characterized in the seeds of the Egyptian cultivars of Fenugreek ‘Giza 2’ and ‘Giza 30’.

**Table 2:**
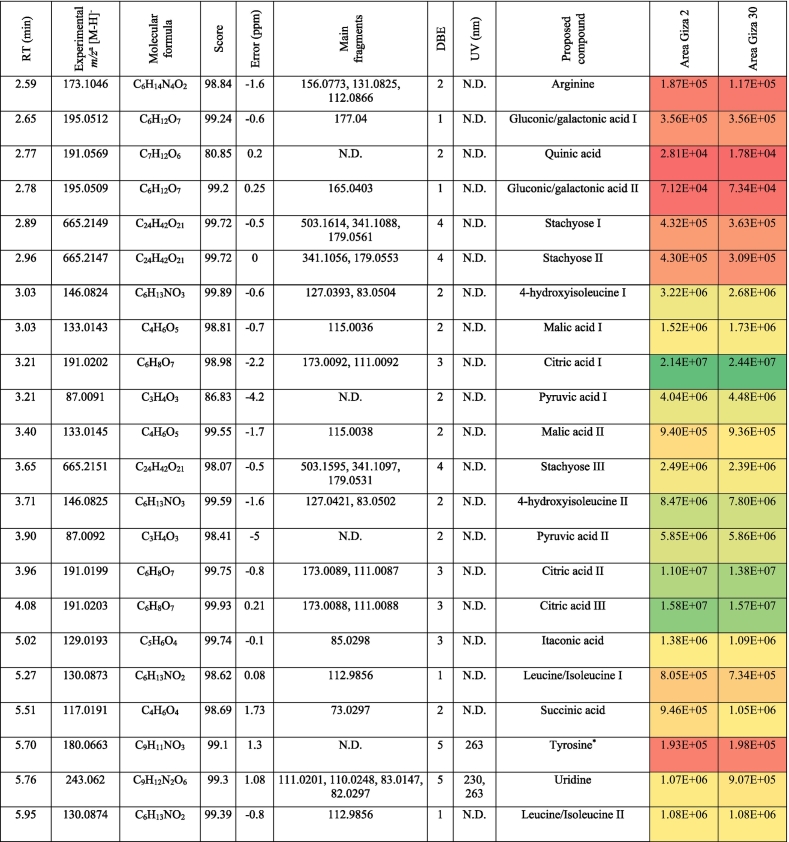

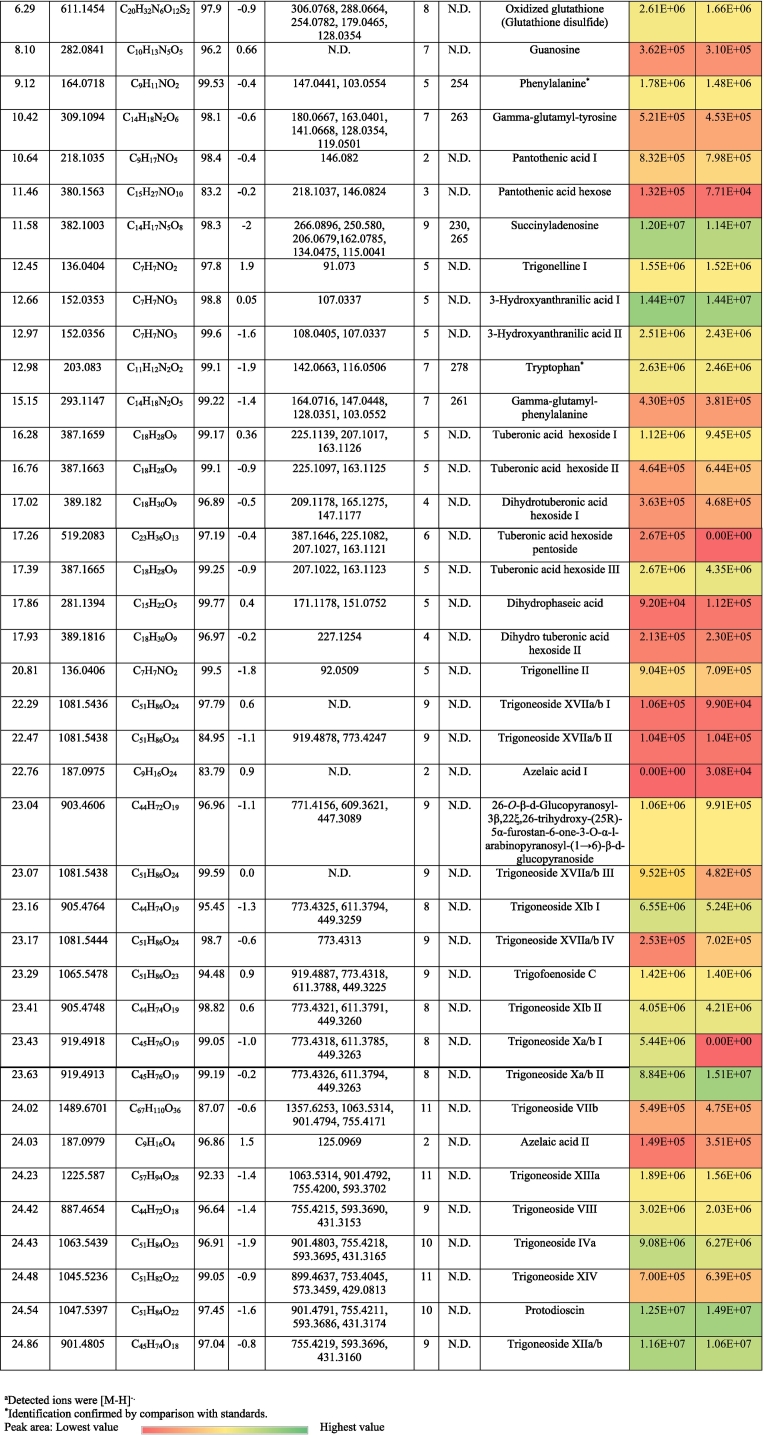
Non-phenolic compounds characterized in the seeds of the Egyptian cultivars of Fenugreek ‘Giza 2’ and ‘Giza 30’.

#### 3.1.1 Phenolic compounds

##### 3.1.1.1 Phenolic acids and coumarins

Eight hydroxybenzoic acid derivatives were annotated as benzoic acid, protocatechuic, and vanillic acid derivatives; most of them were glycosylated. They exhibited the neutral losses of conjugated sugar moieties, if present, followed by decarboxylation (Mekky et al., 2015; Mekky et al., 2021). In this line, Fig. S1a illustrates the fragmentation pattern of hydroxybenzoic acid hexoside. *p*-hydroxybenzoic acid, protocatechuic acid, vanillic acid, and gallic acid were confirmed with authentic standards. It bears noting hydroxybenzoic acid hexoside isomers, hydroxybenzoic acid hexoside pentoside, and vanillic acid hexoside pentoside, were reported in Family Fabaceae (Mekky et al., 2015).

Hydroxycinnamic acids occurred as *p*-coumaric acid and ferulic acid (this only in ‘G30’) alongside five coumaroyl glycosides. While the formers were confirmed with standards, the latter compounds could tentatively be identified by observation of the neutral loss of hexosyl moiety and the appearance of the *p*-coumaric acid ion at *m/z* 163.04 followed by its decarboxylated ion at *m/z* 119.05 (Mekky et al., 2022; Tej et al., 2024) (Fig. S1b).

**Fig. 2:**
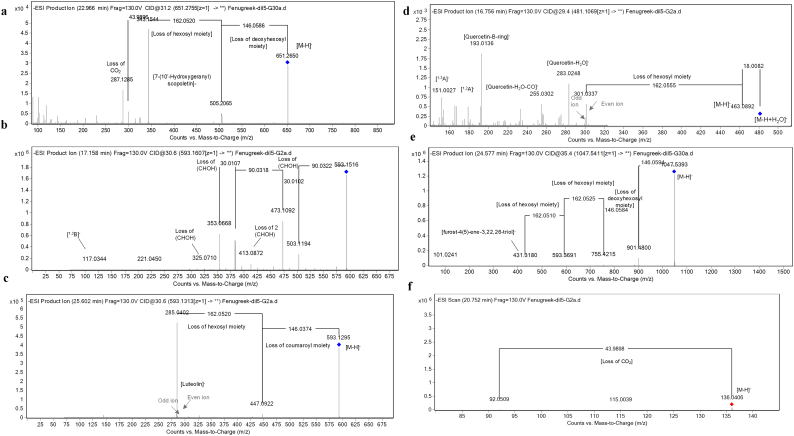
Pattern of fragmentation of (a) 7-(10’-Hydroxygeranyl)scopoletin-10 deoxyhexoside hexoside, (b) apigenin di *C* hexoside III, (c) luteolin *O* coumaroyl hexoside V, (d) quercetin *O* hexoside in hydrated form, (e) protodioscin, (f) trigonelline.

As for coumarins, 7, -(10’-hydroxy geranyl)scopoletin-10 deoxyhexoside hexoside was observed with *m/z* 651.27 expressing the losses of sugar moieties followed by the appearance of the aglycone (*m/z* 325.14) (Fig. 2a). It was described previously in Fabaceae (KNApSAcK-Core-System, 2024; Reaxys, 2024).

##### 3.1.1.2 Flavonoids

‘G2’ and ‘G30’ exhibited the presence of flavonoids as the major class of metabolites with 49 derivatives and more than 50 % of the relative abundance of the total identified metabolites. They were subclassified into flavones (37), flavonols (6), and flavanones (5), and proanthocyanidins (1).

###### 3.1.1.2.1 Flavones

Flavones were the utmost subgroup of flavonoids with 37 derivatives of apigenin and luteolin where their aglycones were confirmed with standards. Chrysin (RT, 29.8 min) was annotated with *m/z* 253.05 and the characteristic ions of *m/z* 225.06 and 151 accounting for [M-H-CO]^-^ and [^1,3^ A]^-^, respectively. With regards to apigenin derivatives, they expressed the characteristic fragment ion 117.03 [^1,3^B]^-^. In this sense, the compound of *m/z* 577.16 (C_27_H_30_O_14_) exhibited the neutral loss of a hexosyl (162 Da) and a deoxyhexosyl (146 Da), indicating *O*-conjugation. Hence, it was tentatively described as apigenin *O*-deoxyhexoside hexoside. The rest of the apigenin derivatives (19) showed the *C*-glycosylation pattern with neutral loss of n CHOH groups of (n × 30 Da). Fig. 2b demonstrates the pattern of fragmentation of *C*-glycosylated flavonoids (Mekky, Abdel-Sattar, Segura-Carretero and Contreras, 2019, Mekky, Abdel-Sattar, Segura-Carretero and Contreras, 2021) (Tables 1, S1). Notably, this is the first report of apigenin di-*C*-pentosides in Fabaceae, although the presence of some isomers has been reported in other plant families (Huang, Guo, Gao, Chen, & Olatunji, 2015; Mekky, Abdel-Sattar, Segura-Carretero and Contreras, 2019, Mekky, Abdel-Sattar, Segura-Carretero and Contreras, 2021). Also, three apigenin derivatives exhibited a neutral loss of 162 Da among the pattern of fragmentation of *C*-glycosylated flavonoids, and they were characterized as apigenin *O-*hexoside *C-*pentoside hexoside, which was described before in *Ceratonia siliqua* (Fabaceae) as schaftoside 4’-*O*-glucoside and isoschaftoside 4’-*O*-glucoside (KNApSAcK-Core-System, 2024) (Tables 1 and S1).

In the same manner, luteolin derivatives portrayed both the *O*- and *C*-glycosylation with the appearance of luteolin ion at *m/z* 285.04 in the former conjugation or the notice of n CHOH groups in the latter one and fragment ions of *m/z* 151 ([^1,3^ A]^-^) and 133 ([^1,3^B]^-^). (Tables 1 and S1). Moreover, luteolin methyl ether glycosides were found with a fragmentation pattern similar to *O*-glycosides along the loss of the methyl moiety. Therefore, glycosylated flavonoids, especially those that present both *C*- and *O*-glycosides, could be a distinctive feature of fenugreeks. Highly interesting was also the characterization of five isomers of luteolin *O*-coumaroylhexoside, characterized by the neutral loss of coumaroyl ion at *m/z* 146.04 as a marker fragment for their identification, and then the hexose moiety (Fig. 2c).

###### 3.1.1.2.2 Flavonols and other flavonoids

Six flavonols were annotated in ‘G2’ and ‘G30’. Quercetin, quercetin 3-*O*-rhamnopyranoside, kaempferol, and kaempferide were confirmed with standards with the presence of fragment ions *m*/*z* 193, 179, and 151 indicating the loss of ring B, fragments [^1,2^ A]^-^, and [^1,3^ A]^-^_,_ respectively (Mekky et al., 2022) (Tables 1 and S1). Fig. 2d portrays the pattern of fragmentation of the hydrated form of quercetin *O*-hexoside with a former step of water loss. Five flavanones were also characterized: pinocembrin, naringenin and their glycosides, eriodictyol *C*-hexoside, and the proanthocyanidin procyanidin A2 were noticed in the ‘G2’ and ‘G30’. Both naringenin and procyanidin A2 were confirmed with standards (Tables 1 and S1).

#### 3.1.2 Non-phenolic compounds

##### 3.1.2.1 Saponins

Saponins were represented by 17 furostanol glycosides. Depending on the pattern of their glycosylation, these compounds showed the neutral losses of pentosyl (132 Da), deoxyhexosyl (146 Da), and hexosyl moieties (162 Da), while remaining the aglycone steroid counterpart. In this sense, four metabolites were annotated as trigoneoside VIIb, trigoneoside XIIIa, trigoneoside IVa, protodioscin, and with a common aglycone of 25 (S/R)furost-5-ene-3β,22ξ,26-triol *m/z* 431 (Tables 2, S2) (Murakami et al., 2000; Pang et al., 2012). Fig. 2e displays the pattern of fragmentation of protodioscin. Besides, trigoneoside VIII, and trigoneoside XIIa/b exhibited similar fragmentation patterns with steroidal counterparts of 25R-5α-furostan-20 (22)-en-2α,3β,26-triol (Shang, Cai, Kadota, & Tezuka, 2001) and (25*R*/*S*)-furost-4-ene-3β22ξ26 triol (Murakami et al., 2000), respectively. Trigoneoside Xa/b I-II, trigoneoside XIb I-II, and trigoneoside XVIIa/b I-IV exhibited the neutral losses of sugar moieties with the steroidal aglycone (25*R*/*S*)-5α-Furostane-2α-3β22ξ26tetraol at *m/z* 449 (Murakami et al., 2000; Pang et al., 2012) whereas trigofoenoside C showed a steroidal counterpart of (25*R*)-22-*O*-methyl-5α-furostane-2α,3β,26-triol at *m/z* 449 (Gupta, Jain, & Thakur, 1986). As for trigoneoside XIV, it showed an aglycone of furost-5,25(27)di-ene-3β,22α,26-triol at *m/z* 429 (Pang et al., 2012). Moreover, the ion of *m/z* 903.46 (C_44_H_72_O_19_) was annotated as 26-*O*-β-d-glucopyranosyl-3β,22ξ,26-trihydroxy-(25*R*)-5α-furostan-6-one-3-*O*-α-L-arabinopyranosyl-(1 → 6)-β-D-glucopyranoside or an isomer, which was described for the first time in Fabaceae (Reaxys, 2024).

##### 3.1.2.2 Alkaloids and other compounds

Two isomers of the alkaloid trigonelline (*m/z* 136.04, C_7_H_7_NO_2_) were observed (Fig. 2f), which is a marker of fenugreek seeds (Singh et al., 2022). Also, ten amino acids were detected, showing the neutral loss of 17 Da (NH_3_) and/or 44 Da (CO_2_) (Mekky et al., 2015; Mekky et al., 2021) (Tables 2, S2). Moreover, dipeptides such as gamma-glutamyl-phenylalanine and gamma-glutamyl-tyrosine were noticed in the studied cultivars, agreeing with their previous description in other seeds (Mekky et al., 2015). It bears noting that oxidized glutathione was also observed complying with its characteristic appearance in other seed types like sesame and linseed (Mekky, Abdel-Sattar, Segura-Carretero and Contreras, 2019, Mekky, Abdel-Sattar, Segura-Carretero and Contreras, 2022). The nucleosides uridine, guanosine, and succinyl adenosine (Fig. S1c) were characterized according to previously published works (Mekky et al., 2015; Mekky et al., 2021). 16 organic acids were tentatively identified, agreeing with previous literature, where decarboxylation and dehydration took place (Mekky et al., 2022; Saeed et al., 2024; Tej et al., 2024) as quinic acid, pyruvic acid, citric acid, and pantothenic acid. Two isomers of azelaic acid were found in ‘G30’, and only one in the other cultivar. Six jasmonate derivatives, three tetrasaccharides, and a sesquiterpenoid, were present with common fragments (Mekky et al., 2015) (Tables 2, S2).

### 3.2 Qualitative and quantitative comparison

**Fig. 3:**
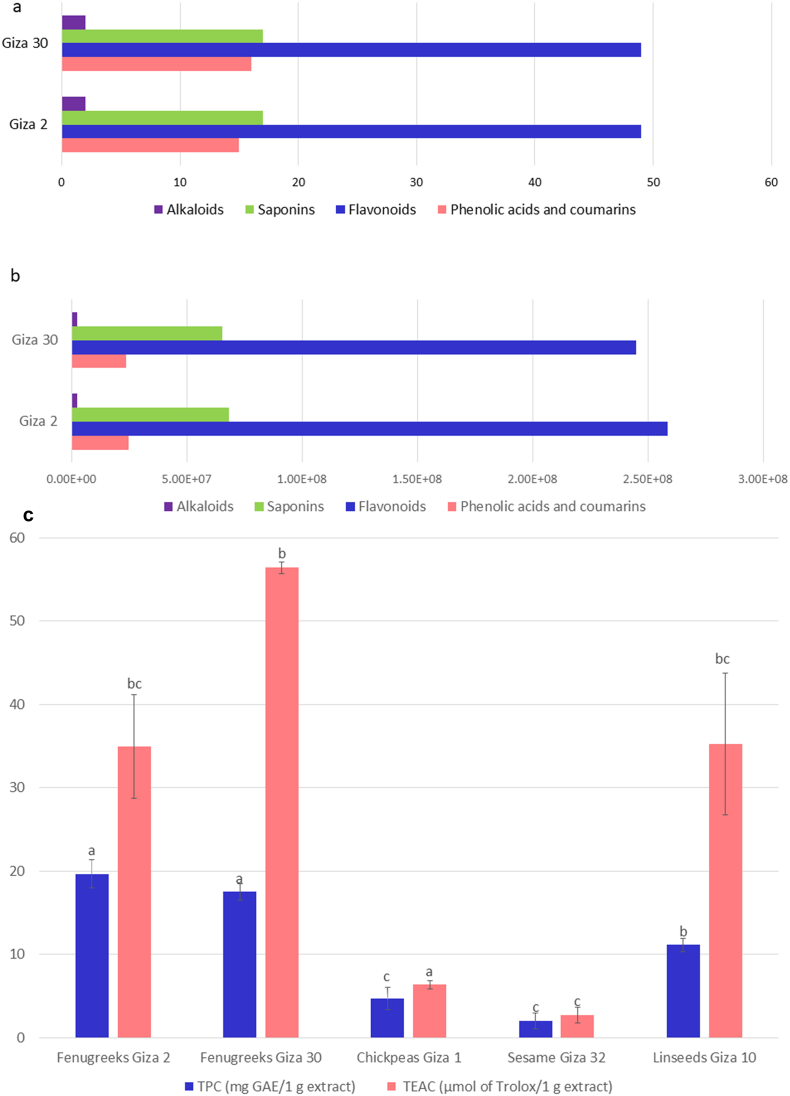
Bioactive metabolites comparison in ‘G2’ and ‘G30’: in terms of (a) metabolites number, and (b) relative abundance, (c) TPC and TEAC (mean ± standard deviation, *n* = 3, *p* < 0.05) for ‘G2’, ‘G30’, chickpeas, sesame, and linseeds.

Qualitatively, ‘G2’ showed the presence of 124 metabolites, while ‘G30’ exhibited 119 metabolites (Fig. 3a). The relative abundance of the bioactive metabolites in ‘G2’, namely phenolic acids, coumarins, flavonoids, saponins, and alkaloids, was beyond that of ‘G30’ (Fig. 3b). The utmost relative abundance of bioactive metabolites in ‘G2’ agrees with the TPC results. Where the TPC of ‘G2’ and ‘G30’ were 19.7 ± 2 and 17.5 ± 1 mg of GAE/g fenugreek seeds extract, respectively, which is in the range of other works (Kaviarasan, Naik, Gangabhagirathi, Anuradha, & Priyadarsini, 2007; Kumar, Anand, Singsit, Khanum, & Anilakumar, 2013). About the TPC of other geographically related seeds, the TPC of fenugreek seeds was higher than those from cultivar ‘Giza 1’ of chickpeas (Fabaceae), cultivar ‘Giza 32’ of sesame (Pedaliaceae), and cultivar ‘Giza 10’ of linseeds (Linaceae) (Fig. 3c).

### 3.3 Antioxidant activity and bioactivity related to fenugreek characteristic seed metabolites

TEAC values for ‘G2’ and ‘G30’ were measured to be 35 ± 6 and 56.4 ± 0.7 μmol TE/g fenugreek seed extract (Fig. 3c). It bears noting that (Hameed et al., 2019) conducted a study on fenugreeks obtained from Faisalabad, where the aqueous and methanolic extracts exhibited TEAC values of 42.7 ± 0.3 and 55.7 ± 0.7 μmol TE/g extract, respectively. Moreover, (Ghoora, Haldipur, & Srividya, 2020) the TEAC value of fenugreek microgreens 13.4 ± 0.0 μmol TE/g and hence the obtained results were in the range of the aforementioned studies. The phenolic composition of fenugreek seeds is related to their notable antioxidant activity, particularly due to the significant presence of flavonoids (Materska, 2015). Flavonoids accounted for 91.3 % and 91.1 % of the total phenolic metabolites in ‘G2’ and ‘G30’, respectively. The antioxidant capacity may be enhanced by the presence of *C*-glycoside of flavones in particular apigenin derivatives *viz*.*,* apigenin di-*C*-hexoside I-IV, apigenin *C*-pentoside *C*-hexoside I-VI, and the first presence of apigenin di *C* pentoside I-III in Fabaceae where metal chelation sites and hydroxyl groups are free (Materska, 2015; Mekky et al., 2019). Moreover, the higher levels of organic acids such as citric acid and pyruvic acid isomers found in ‘Giza 30’ may potentially enhance the antioxidant activity through a synergistic effect in other studies (Quiroga, Nepote, & Baumgartner, 2019; Wang et al., 2007). The existence of reduced glutathione can be inferred from the detection of oxidized glutathione (GSSG), which has the potential to undergo oxidation during analysis and may also influence antioxidant properties (Herzog et al., 2019; Mekky et al., 2019).

Fenugreeks had a greater antioxidant capacity in comparison to other seeds, even those belonging to the same legume family such as chickpeas. The richness of ‘G2’ and ‘G30’ with bioactive metabolites may explain the higher antioxidant activity of fenugreek compared to other seeds of regional cultivars. Besides the antioxidant activity, the presence of furostanol saponins with a predominance of trigoneoside Xa/b I-II, trigoneoside IVa, protodioscin, and trigoneoside XIIa/b in the extracts of fenugreek seeds could attribute antidiabetic potential in accordance with the patent of (Goel, 2010) where furostanol-rich saponin extract exhibited a significant antidiabetic action in a rat model as well as in a clinical trial. The alkaloid trigonelline has also been related to the antidiabetic, antimigraine, sedative, memory-improving, antiviral, antibacterial, and anti-tumor activities of fenugreek (Zhou, Chan, & Zhou, 2012). A recent work (Alsuliam et al., 2022) portrayed in a diabetic rats model, fenugreek seeds aqueous extracts and galactomannan-rich fraction at 500 mg/kg/each reduced islet of Langerhans mass loss, diabetic nephropathy, and liver damage. Additionally, they reduced reactive oxygen species (ROS), lipid peroxides, tumor necrosis factor α (TNF-α), interleukin 6 (IL-6), and nuclear factor kappa B p65 (NF-κB p65) levels.

Also, the study conducted by (Hosseini et al., 2023) exhibited that swimming and fenugreek 80 % ethanolic extract (100 mg/kg) reduced glycemic indices (serum glucose, insulin, and insulin resistance) and lipid profile (total cholesterol, low-density lipoprotein cholesterol, very low-density lipoprotein cholesterol, and triglycerides) while increasing high-density lipoprotein cholesterol.

It is clear that the richness of such bioactive metabolites as phenolic acids, flavonoids, saponins, glutathione, and alkaloids justifies the use of fenugreek seeds in folk medicines and their incorporation in nutraceuticals and dosage forms. Comprising a wide range of phytoconstituents, fenugreeks display a multitude of pharmacological effects compared to other plants. In this sense, Table 3 summarizes the antioxidant, antidiabetic, and anti-inflammatory biological potentials of the metabolites characterized in ‘Giza 2’ and ‘Giza 30’ fenugreek cultivars.

**Table 3:** Biological potentials of metabolites characterized in fenugreek cultivars ‘Giza 2’ and ‘Giza 30’.

Bioactive compound	Biological activity	References
Apigenin and related glycosides	Antioxidant, anti-inflammatory	(Materska, 2015; Mekky et al., 2019; Singh et al., 2022)
Quercetin, kaempferol and related glycosides	Antidiabetic, alleviative diabetic complications, anti-inflammatory, antioxidant	(Abouzed et al., 2018; Mekky et al., 2015)
Phenolic acids	Antidiabetic, alleviative diabetic complications, anti-inflammatory, antioxidant	(Abouzed et al., 2018; Tej et al., 2024)
Furostanol saponins	Antidiabetic, alleviative diabetic complications	(Goel, 2010; Singh et al., 2022)
Glutathione	Antioxidant, anti-inflammatory	(Herzog et al., 2019)
Trigonelline	Antidiabetic, alleviative diabetic complications, anti-inflammatory, antioxidant	(Yao et al., 2020; Zhou et al., 2012)
Citric acid and pyruvic acid	Antioxidant, anti-inflammatory	(Quiroga et al., 2019; Wang et al., 2007)

**Fig. 4:**
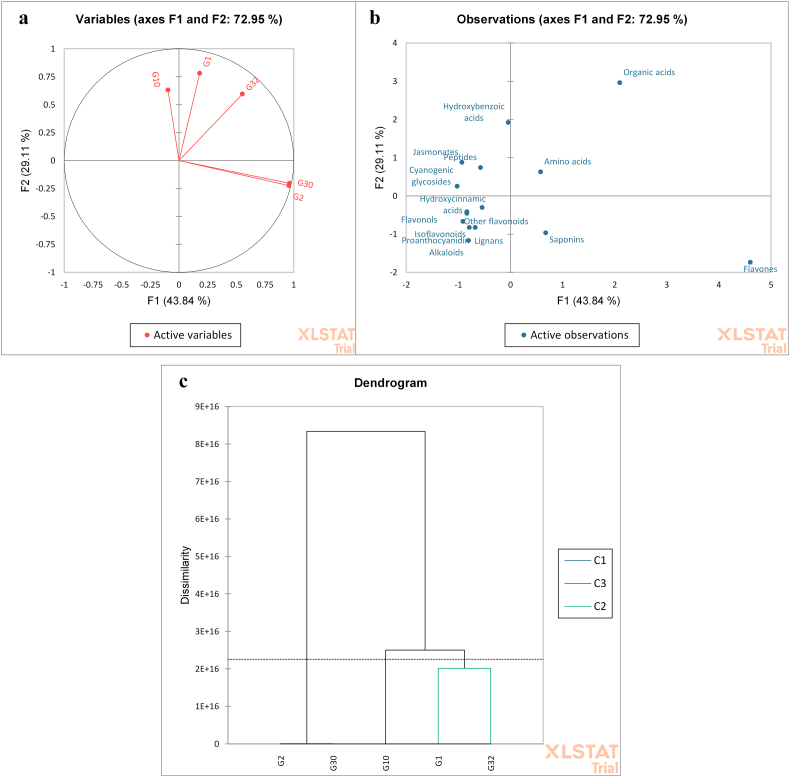
(a) PCA score plot and (b) loading plot. (c) HCA dendrogram of the Egyptian cultivars of fenugreek ‘Giza 2, G2’, ‘Giza 30, G30, chickpea ‘Giza 1, G1’, sesame ‘Giza 32, G32’, and linseed ‘Giza 10, G10’.

Furthermore, a chemometric analysis based on metabolites subclasses relative amounts were employed (Mekky et al., 2022; Sweilam, Abd El Hafeez, Mansour, & Mekky, 2024) to discriminate the aforementioned geographically related seeds (Mekky et al., 2015; Mekky, Abdel-Sattar, Segura-Carretero and Contreras, 2019, Mekky, Abdel-Sattar, Segura-Carretero and Contreras, 2022) by principle component analysis (PCA) and hierarchical cluster analysis (HCA). In this context, the score plot of the PCA figured out that the first three components accounted for 83.4 % of the total data variance. Both fenugreek cultivars, ‘Giza 2’ and ‘Giza 30’, were closely related to each other in the same quadrant and discriminated from the other seeds. Moreover, chickpea seed cultivar ‘Giza 1’ and sesame seed cultivar ‘Giza 32’ were closely related to each other, being in the same quadrant, whereas linseed cultivar ‘Giza 10’ was segregated from all the other seeds (Fig. 4a). Besides, the loading plot exhibited that metabolites subclasses relative amounts contributed to separating the aforementioned seeds where some discriminative markers were highlighted as the saponin and flavones contents of fenugreeks (Table 1, Table 2) as well as the cyanogenic glycosides and peptides in linseeds (Fig. 4b). As for HCA, it revealed that fenugreek cultivars ‘Giza 2’ and ‘Giza 30’ were distant from other cultivars, being clustered in one main separate group. Linseeds ‘Giza 10’ were related to chickpea ‘Giza 1’ and sesame ‘Giza 32’ being in one main cluster, while the latter two were closely related to a subcluster (Fig. 4c).

## 4 Conclusions

The present work provides a comprehensive metabolomic profiling of two fenugreek cultivars, identifying a wide range of 126 phytochemicals, including a substantial amount of phenolics and other bioactive components.” The characterized metabolites in the fenugreek seed extracts played a significant role in their remarkable antioxidant ability, surpassing that of other geographically related seeds. Furthermore, the considerable occurrence of trigonelline and furostanol saponins indicates the potential antidiabetic effects of the seeds. The present study highlights the potential of fenugreek seed extracts as highly valued resources for the nutraceutical, supplement, and food industries. The abundant antioxidants in fenugreeks could be utilized to create functional foods and drinks that have improved oxidation resistance. Moreover, the antidiabetic characteristics of fenugreek could guide the refinement of specific dietary supplements for diabetic and/or pre-diabetic patients. Future studies should prioritize examining the bioavailability of these metabolites in human bodies to evaluate their possible therapeutic effectiveness. Clinical trials are also necessary to confirm the results obtained in laboratory and determine the most effective dosage and administration methods for different purposes. Moreover, investigating the mechanisms that underlie the biological activities of these phytochemicals could offer valuable insights for advancing innovative therapeutic strategies.

## Funding

The authors acknowledge financial support from the Researchers Supporting Project number (RSP2024R344), King Saud University, Riyadh, Saudi Arabia. This work was supported by the International Cooperation Cell ICC06 under the Erasmus Mundus – Al Idrisi II programme “scholarship scheme for exchange and cooperation between Europe and North Africa”. M.d.M.C. thanks the Ministry of Science and Innovation of Spain for the Ramón y Cajal grant (RYC2020-030546-I/ AEI/ 10.13039/501100011033) and the European Social Fund.

## CRediT authorship contribution statement

**Reham Hassan Mekky:** Writing – original draft, Validation, Investigation, Formal analysis, Data curation. **Essam Abdel-Sattar:** Writing – review & editing, Supervision. **Maha-Hamadien Abdulla:** Validation, Methodology, Funding acquisition. **Antonio Segura-Carretero:** Writing – review & editing, Supervision. **Khayal Al-Khayal:** Writing – review & editing, Writing – original draft, Validation, Methodology. **Wagdy M. Eldehna:** Writing – review & editing, Writing – original draft, Formal analysis. **María del Mar Contreras:** Writing – review & editing, Supervision, Methodology, Investigation.

## Declaration of competing interest

The authors declare that they have no known competing financial interests or personal relationships that could have appeared to influence the work reported in this paper.

## Data Availability

The data are contained within the article or supplementary materials section.

## Appendix A Supplementary data

Supplementary material 1Table S1. Phenolic compounds characterized in the seeds of the Egyptian cultivars of Fenugreek ‘Giza 2’ and ‘Giza 30’., Table S2. Non-phenolic compounds characterized in the seeds of the Egyptian cultivars of Fenugreek ‘Giza 2’ and ‘Giza 30’., **Figure S1:** Pattern of fragmentation of  a) hydroxybenzoic acid hexoside I, b) *p*-coumaric acid hexoside, c) succinyladenosine. Image 1

Supplementary material 2 Image 2
