# Supplementary material for: Metabolic profiling and antioxidant activity of fenugreek seeds cultivars ‘Giza 2’ and ‘Giza 30’ compared to other geographically-related seeds
Source: Food Chem X. 2024 Sep 7;24:101819. doi: 10.1016/j.fochx.2024.101819 (PMC11426063; doi:10.1016/j.fochx.2024.101819)
Supplement: Supplementary file 2 — Supplementary material 2 [file mmc2.docx]

Supplementary Materials

**Metabolic Profiling and Antioxidant Activity of Fenugreek Seeds Cultivars 'Giza 2' and 'Giza 30' Compared to Other Geographically-Related Seeds** Reham Hassan Mekky^1,2,*^, Essam Abdel-Sattar^3^, Maha-Hamadien Abdulla^4^, Antonio Segura-Carretero^2,5^, Khayal Al-Khayal^4^, Wagdy M. Eldehna^6,7*^, and María del Mar Contreras^8,*^

*^1^Department of Pharmacognosy, Faculty of Pharmacy, Egyptian Russian University, Badr City, Cairo-Suez Road, 11829, Cairo, Egypt*

*^2^ Research and Development Functional Food Centre (CIDAF), Bioregiόn Building, Health Science Technological Park, Avenida del Conocimiento s/n, 18016, Granada, Spain*

*^3^ Department of Pharmacognosy, Faculty of Pharmacy, Cairo University, El Kasr El-Aini Street, 11562, Cairo, Egypt*

*^4^Department of Surgery, College of Medicine, King Saud University, Riyadh, Saudi Arabia*

*^5^ Department of Analytical Chemistry, Faculty of Sciences, University of Granada, Avenida Fuentenueva s/n, 18071, Granada, Spain*

*^6^Department of Pharmaceutical Chemistry, Faculty of Pharmacy, Kafrelsheikh University, Kafrelsheikh, P.O. Box 33516, Egypt*

*^7^Department of Pharmaceutical Chemistry, Faculty of Pharmacy, Pharos University in Alexandria; Canal El Mahmoudia St., Alexandria 21648, Egypt*

*Correspondence: [reham-mekky@eru.edu.eg](mailto:reham-mekky@eru.edu.eg) (R.H.M.); [wagdy2000@gmail.com](mailto:wagdy2000@gmail.com) (W.M.E.); [mcgamez@ujaen.es](mailto:mcgamez@ujaen.es) (or [mar.contreras.gamez@gmail.com](mailto:mar.contreras.gamez@gmail.com), M.d.M.C.)


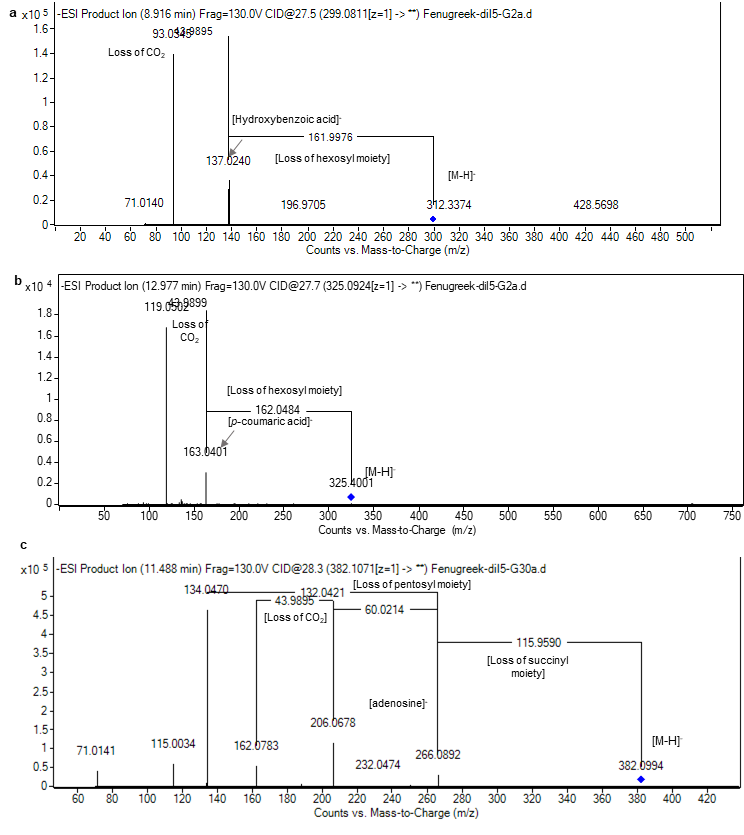


**Figure S1:** Pattern of fragmentation of a) hydroxybenzoic acid hexoside I, b) *p*-coumaric acid hexoside, c) succinyladenosine
